# External validation of existing dementia prediction models on observational health data

**DOI:** 10.1186/s12874-022-01793-5

**Published:** 2022-12-05

**Authors:** Luis H. John, Jan A. Kors, Egill A. Fridgeirsson, Jenna M. Reps, Peter R. Rijnbeek

**Affiliations:** 1 Department of Medical Informatics, Erasmus University Medical Center, Dr. Molewaterplein 40, 3015 GD Rotterdam, The Netherlands; 2 Janssen Research and Development, 1125 Trenton Harbourton Rd, NJ 08560 Titusville, USA

**Keywords:** Patient-level prediction, Prognostic model, External validation, Transportability, Dementia, Alzheimer

## Abstract

**Background:**

Many dementia prediction models have been developed, but only few have been externally validated, which hinders clinical uptake and may pose a risk if models are applied to actual patients regardless. Externally validating an existing prediction model is a difficult task, where we mostly rely on the completeness of model reporting in a published article.

In this study, we aim to externally validate existing dementia prediction models. To that end, we define model reporting criteria, review published studies, and externally validate three well reported models using routinely collected health data from administrative claims and electronic health records.

**Methods:**

We identified dementia prediction models that were developed between 2011 and 2020 and assessed if they could be externally validated given a set of model criteria. In addition, we externally validated three of these models (Walters’ Dementia Risk Score, Mehta’s RxDx-Dementia Risk Index, and Nori’s ADRD dementia prediction model) on a network of six observational health databases from the United States, United Kingdom, Germany and the Netherlands, including the original development databases of the models.

**Results:**

We reviewed 59 dementia prediction models. All models reported the prediction method, development database, and target and outcome definitions. Less frequently reported by these 59 prediction models were predictor definitions (52 models) including the time window in which a predictor is assessed (21 models), predictor coefficients (20 models), and the time-at-risk (42 models). The validation of the model by Walters (development c-statistic: 0.84) showed moderate transportability (0.67–0.76 c-statistic). The Mehta model (development c-statistic: 0.81) transported well to some of the external databases (0.69–0.79 c-statistic). The Nori model (development AUROC: 0.69) transported well (0.62–0.68 AUROC) but performed modestly overall. Recalibration showed improvements for the Walters and Nori models, while recalibration could not be assessed for the Mehta model due to unreported baseline hazard.

**Conclusion:**

We observed that reporting is mostly insufficient to fully externally validate published dementia prediction models, and therefore, it is uncertain how well these models would work in other clinical settings. We emphasize the importance of following established guidelines for reporting clinical prediction models. We recommend that reporting should be more explicit and have external validation in mind if the model is meant to be applied in different settings.

**Supplementary Information:**

The online version contains supplementary material available at 10.1186/s12874-022-01793-5.

## Key points

- Many dementia prediction models have been developed, but only few have been externally validated, which may limit clinical uptake.
- We assess whether reported information on existing dementia prediction models allows for external validation.
- We externally validate three of the dementia prediction models across a network of observational databases, including the original development databases.

## Background

Dementia is an umbrella term to describe various illnesses that affect cognition and may lead to mental degradation. Early diagnosis of individuals at high risk of dementia allows for improved care and risk-factor targeted intervention [1]. Many patient-level prediction models for identifying individuals who are at risk of dementia have been developed [2–4].

Earlier models were mostly developed on data from cohort studies where data were recorded during health checkups using a variety of questionnaires and cognition tests [5–10]. In recent years models have increasingly been developed on observational health data [11–16]. These routinely collected data from administrative claims and electronic health records are considered to enhance a model’s applicability at the point of care [15]. Although observational health data generally do not include known predictive variables such as education level, cognitive test results and genetic information [15], various studies have shown good internal validation performance when developing models on this kind of data. Notable examples are Walters et al. who developed dementia prediction models using electronic health record data from the THIN database, and Albrecht et al. who developed predictive models for Alzheimer’s disease and related dementias (ADRD) using administrative claims data from the OptumLabs Data Warehouse [14, 15].

However, the systematic reviews of Hou et al. and Goerdten et al. conclude that although many dementia risk prediction models have been developed, only a handful of them have been externally validated [3, 4]. External validation assesses a model’s reliability for clinical use in external data sources that have not been used for model development. A lack of external validation can lead to a plethora of proposed models with little evidence about which are reliable and under what circumstances [17].

External validation can be a cumbersome process due to the difficulty of retrieving a prediction model, e.g., retrieving cohort and predictor definitions, or coefficient values from a published manuscript. We hypothesize that successful model retrieval largely depends on completeness of model reporting. Insufficient reporting may prevent efficient and large-scale external validation, potentially resulting in small clinical uptake of published models [18].

In this study, we aim to externally validate existing dementia prediction models. To that end, we define model reporting criteria, review published models, and externally validate three selected models using routinely collected health data from administrative claims and electronic health records.

## Methods

### Article selection

Our literature search for existing dementia prediction models was based on the search query presented in a systematic review on dementia risk prediction modelling by Tang et al. from 2015 [2]. The search interval was extended from 1 to 2011 to 31 December 2020. MEDLINE, Embase, Scopus and ISI Web of Science were originally searched using combinations of the following terms and mapped to Medical Subject Headings (MeSH): “dementia”, “Alzheimer disease”, “Alzheimer and disease”, “predict”, “develop”, “incident”, “sensitivity”, “specificity”, “ROC” and “area under the curve” [2]. For our query we added the following terms: “c statistic”, “concordance statistic”. Only articles published in English were considered.

Articles were included if they met the following criteria: (1) the sample was population-based; (2) the risk model predicts the risk of dementia in non-demented individuals; (3) measurements of discrimination are provided, e.g., the area under the receiver operating characteristic curve (AUROC) or c-statistic.

### Reporting criteria

**Fig. 1:**
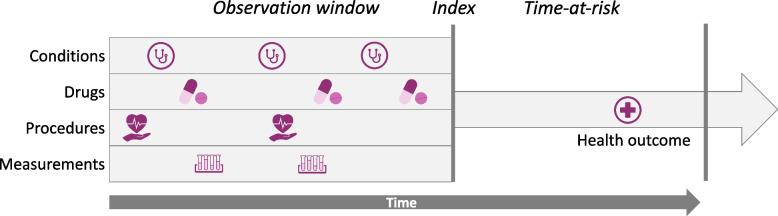
Patient-level prediction time windows and index date

This study does not develop or propose a prediction model, but merely applies existing models. If the prediction models to be applied are not presented in the form of a calculator, e.g., as a nomogram or chart score, it is necessary to retrieve them from the accompanying documentation, such as the research paper and supplemental material. The criteria that a study must report for model validation on external data to be feasible are presented in Table 1 and can be directly inferred from our prediction approach (Fig. 1). Among a population at risk, we predict which patients at a defined moment in time (the index) will experience some outcome during a time-at-risk. Prediction is done using only information about the patients in an observation window prior to the index.

Reporting criteria can be broadly categorized into population settings and statistical analysis settings (Table 1) [19].

**Table 1:** Model reporting criteria that prediction studies should report to enable external validation

Category	Reporting criteria	Description
Population settings	Target population definition	Definition or description of the population for which predictions are made.
	Index date	Date at which a patient qualifies for inclusion in the target population.
	Time-at-risk	Time window in which a model’s predictions are valid relative to the index date.
	Outcome definition	Definition or description of the outcome to be predicted during the time-at-risk.
Statistical analysis settings	Prediction method	Prediction methods in this study are limited to logistic regression and Cox proportional hazard for predicting a binary outcome.
	Predictor definitions	Predictor descriptions or definitions in terms of data source codes.
	Predictor time window	Time window in which the predictor is assessed. In a special case, a predictor can be assessed in a time window all time prior to index, often reported as “a history of …” using all available prior data of a person.
	Model specifications	The prediction model, e.g., parameters to construct the model given a prediction method. Alternatively, a risk calculator or nomogram could be reported. We also distinguish here between fully and partially specified models. For example, if no intercept is reported in the case of a logistic regression model, we are still able to construct a simple risk stratification model using only coefficient values. However, this method does not consider the baseline risk of the original model and (re-)calibration will not be assessed.

### Data sources

For external validation, we selected a diverse set of electronic health record (EHR) and claims observational databases from different countries (Table 2).

IBM MarketScan Medicare Supplemental Database (MDCR) includes data from the health services of retirees in the United States with Medicare supplemental coverage through employer-sponsored plans. Optum De-Identified Clinformatics Data Mart – Socio-Economic Status (OPSES) Database is derived from administrative health claims for members of large commercial and Medicare Advantage health plans in the United States. The Iqvia Disease Analyzer Germany (IQGER) database consists of mostly primary care physician data collected from German practices and medical centers for all ages. Optum de-identified Electronic Health Record Dataset (OPEHR) represents longitudinal EHR data derived from dozens of healthcare provider organizations in the United States. The Clinical Practice Research Datalink (CPRD) is a governmental, not-for-profit research service consisting of data collected from UK primary care for all ages. The Integrated Primary Care Information (IPCI) database is a Dutch database containing the complete medical record of patients provided by around 350 general practitioners (GPs) geographically spread over the Netherlands [20]. Iqvia Medical Research Database (IMRD), incorporating The Health Improvement Network (THIN), is a longitudinal patient database collected from primary care practices in the UK.

All data sources have been mapped to the Observational Medical Outcome Partnership (OMOP) Common Data Model (CDM) version 5, which provides a standardized data structure and vocabulary [21].

**Table 2:** Data sources selected for external validation of selected dementia prediction models

Database	Acronym	No. of patients (million)	Country	Data type
IBM MarketScan® Medicare Supplemental Database	MDCR	10	United States	Claims
Iqvia Germany DA	IQGER	30	Germany	GP, EHR
Optum’s de-identifed Clinformatics® Data Mart Database	OPSES	85	United States	Claims
Optum® de-identified Electronic Health Record dataset	OPEHR	94	United States	EHR
Clinical Practice Research Datalink	CPRD	13	United Kingdom	GP
Integrated Primary Care Information	IPCI	2.5	Netherlands	GP
Iqvia Medical Research Database (incorporating THIN)	IMRD	18	United Kingdom	GP

### Model selection for external validation

From the reviewed studies, we select models that were developed on one of our included data sources (Table 2). We validate these models on their original development database, which allows us to approximate quality of model reporting. In the optimal case the same discrimination performance as in the research paper should be achieved. A significantly lower performance could indicate poor model reporting. The performance of a model on its original development database will be referred to as “round-trip” performance. In addition, the selected models were externally validated on the remaining data sources.

If only predictor coefficients are reported, we can construct a risk stratification model that identifies high/low risk patients but does not assign an absolute risk estimate [22]. This is achieved by scaling the maximum achievable score of ${\theta }^{T}X$, $\theta$ being the coefficients, or points per predictor, and $X$ being the predictors, with values between 0 and 1. Because there is no parameter that indicates baseline risk of the development population, we cannot assign a risk estimate and will not assess calibration for this kind of model.

For external validation we use the standardized patient-level prediction framework (PLP), which was developed by the Observational Health Data Science and Informatics (OHDSI) network [19]. This framework enables the development of analysis packages in R that can be shared across sites that have access to data sources OMOP CDM. Our validation packages are populated on-site through computer-executable cohort and predictor definitions using SQL queries. The patient-level-prediction framework is based on best practices proposed by the Prognosis Research Strategy (PROGRESS) and follows the recommendations of the Transparent Reporting of a Multivariable Prediction Model for Individual Prognosis or Diagnosis (TRIPOD) statement [23, 24].

### Performance evaluation

External validation of a prediction model typically involves quantifying a model’s discrimination and calibration performance.

A model’s predicted risks must discriminate well between those participants who will and will not have the outcome of interest. Discrimination is generally reported by the area under the receiver operating characteristic curve (AUROC) or when censoring is considered by the concordance statistic (c-statistic), which in practice will take the same value for a binary prediction problem [25]. We will use the AUROC to measure discrimination performance, which is computed as the area under the receiver operating curve, the plot of sensitivity vs. 1-specificity as the value of the cut-off point moves from 0 to 1. To build the receiver operating characteristic (ROC) curves we use the pROC R-package, which also includes functions for computing confidence intervals and methods for smoothing and visualizing ROC curves [26].

Calibration examines the agreement between predicted and observed risks. In literature, calibration was found to be assessed far less often than discrimination, despite the risk of poorly calibrated prediction models being misleading and potentially harmful for clinical decision-making [27]. Various forms of calibration exist. A prediction model is moderately calibrated if, among patients with the same predicted risk, the observed event rate equals the predicted risk [28]. Mean-calibration evaluates whether the observed event rate equals the average predicted risk. However, mean calibration is considered insufficient as sole criterion, as it is satisfied when the predicted risk for each patient would equal the true event rate [28]. Weak calibration is given for a calibration intercept of 0 and a calibration slope of 1. Although not considered flexible, weak calibration can be useful for external validation as calibration intercept and slope can provide a concise summary of potential calibration problems [28].

only require the averaged predicted risk, and weakly-calibrated prediction models only requires the average prediction effects.

Calibration can be visualized graphically in various ways, for example by plotting observed versus predicted risks across deciles of predicted risk or age groups. For this study, we decided on the latter method using age groups.

Transported models may also benefit from re-calibration so that predicted risk better matches the proportion of subjects that actually have the dementia outcome in the external data source. We will use slope and intercept re-calibration. To assess the relative improvement of a model through recalibration, we will assess E_avg_, a single value metric, which is the average absolute difference between observed and predicted probabilities [29].

## Results

### Model reporting

The inclusion criteria of our literature search were met by 35 studies, which described a total of 59 prediction models [5, 7–16, 30–53]. Table 3 summarizes the number of models that fulfil the reporting criteria. Refer to supplemental material in Appendix A for the full literature review.

**Table 3:** Reporting criteria for included dementia prediction models

Category	Reporting criteria	No. of models (%)
Population settings	Target population definition	59 (100)
	Index date	22 (37)
	Time-at-risk	42 (71)
	Outcome definition	59 (100)
Statistical analysis settings	Prediction method	59 (100)
	Predictor definitions	52 (88)
	Predictor time window	21 (36)
	Model specifications: Full model	9 (15)
	Model specifications: Partial model	17 (29)

Criteria that were reported by all included studies were the target population definition, the outcome definition, and the prediction method. Various prediction methods were used, including Cox proportional hazard, single tests, logistic regression, linear discrimination analysis, competing risk regression, disease state index, random forest, and support vector machine. Most frequently reported prediction methods were Cox proportional hazard (13 studies, 21 models) and logistic regression (8 studies, 14 models).

Frequently reported criteria were the time-at-risk (71% of the models) and the predictor definitions (88% of the models). Most reported time-at-risk was between three and five years. Studies that did not explicitly state the time-at-risk or predicted over the full follow-up time of a patient were considered to not report this criterion.

Rarely reported criteria include the index date, the predictor time window, and the full model specifications. Non-demographic predictors were most commonly measured in a time window between one year and five years before index.

Of the included studies, three fulfilled all nine reporting criteria for a total of seven proposed models [30]. The median number of reported criteria across all included models was five.

### Externally validated models

We selected one of the seven fully reported models, Walters’ Dementia Risk Score for persons aged 60–79, for validation and dismissed the other six for various reasons: Walters et al. did not endorse their second model aimed at persons aged 80–95 for clinical use due to low discrimination performance [15]; four models used detailed education variables (0 to 5 years of primary school, Vocational school certificate, French junior-school diploma, French high school diploma, Graduate studies) that were unavailable in the validation databases [32]; one model was developed on data from a prospective cohort study, Adult Changes in Thought (ACT), which is currently not available in the OMOP CDM format [30]. Of the partially reported models, there were two for which the development data and predictors were available in the OMOP CDM, and for which missing criteria, such as the baseline hazard and time-at-risk could be left out or approximated under reserve. Therefore, we selected the following three models for external validation summarized in Table 4: (1) Walters’ Dementia Risk Score which predicts 5-year risk of first recorded dementia diagnosis among patients aged 60–79 using a Cox proportional hazard model and was developed on THIN/IMRD [15]; (2) Mehta’s RxDx-Dementia Risk Index which predicts risk of incident dementia among patients diagnosed with type 2 diabetes mellitus and hypertension using a Cox proportional hazard model developed on CPRD [16]; and (3) Nori’s ADRD prediction model which predicts Alzheimer’s disease and related dementias (ADRD) among patients aged 45 and older using a logistic regression model developed on OptumLabs [13].

**Table 4:** Summary of validated prediction models

	Walters models	Mehta model	Nori model
Development database	THIN	CPRD	OptumLabs
Population	Patients aged 60–79	Patients diagnosed with type 2 diabetes mellitus and hypertension	Patients aged 45 and older
Outcome	5-year risk of first recorded dementia diagnosis	Risk of incident dementia	Risk of Alzheimer’s disease and related dementias (ADRD)
Prediction method	Cox proportional hazard	Cox proportional hazard	Logistic regression
Predictor summary	15 predictors including sociodemographic measures, health status measurements, medical diagnoses, prescription medication	26 predictors including demographic measures, medical diagnoses, medical procedures	50 predictors including demographic measures, medical diagnoses, prescription medication, medical procedures

Of the externally validated models, the Mehta model did not report the baseline hazard and the time-at-risk, and the Nori model did not report the time-at-risk. Because missing information could not be provided by authors, we decided to use a 5-year time-at-risk as used by the Walters model and many of the other reviewed models.

#### Walters model

The Walters model was found to fulfil all reporting criteria defined in Table 1. It was developed on the THIN database and had several notable modeling decisions. For example, during development data imputation has been used for various numeric variables. Of the six imputed variables (smoking, height, total cholesterol, HDL cholesterol, systolic blood pressure, and weight) only smoking remained in the final model. In the validated model the smoking status is imputed by assuming that patients that have neither a code for “smoker” nor “ex-smoker” are considered “non-smokers”. This demonstrates a general shortcoming of observational data, as the absence of a code does not guarantee the absence of a condition, drug, or in this case smoking, and the code may simply not have been recorded despite the patient being a smoker or ex-smoker.

The Walters models uses a variable called “social deprivation score”, which ranges from 1 to 5 indicating social deprivation. The information in this variable has been established through a linkage of the UK postal (zip) code recorded in patient notes to UK Population Census data. However, this linkage is no longer available, unlikely to exist in other databases across the world, and establishing the linkage may not be possible or feasible.

The index date (and start of follow-up) of the Walters model is the latest of four entry events: (1) 1 January 2000, (2) when the individual turned 60, (3) one year following new registration with a THIN practice, and (4) one year after the practice met standard criteria for accurate recording of deaths, consultation, health measurements and prescribing. Only the index date of a patient turning 60 could be fully replicated. The start of follow-up on 1 January 2000 was not applicable to any of the data sources as it lies too far in the past. The remaining two index events are THIN-specific and could not be replicated in other databases, including IMRD. To still make the cohort compatible with the remaining data sources, we added an entry event defined as the latest visit before 1 January 2014 for persons aged 60–79. Since the included data sources could provide records until the end of 2018, we ensure that all persons are eligible for full 5-year risk follow-up as required by the model specifications. Visits are suitable for defining index dates, because they indicate interaction with a healthcare provider that may be qualified to apply a model and interpret its predictions.

The paper mentions that Read codes were used for development, which is a hierarchical coding system that maps onto ICD-10 codes. The authors provide literal names of predictors, for which the corresponding code could be determined by us.

#### Mehta model

The research paper does not report the full model, which is a Cox proportional hazard model. While the coefficients are reported, the baseline hazard and the time-at-risk are missing. We have contacted the authors of this study, but they were unable to provide us with this information. We are still able to validate the model for an estimated time-at-risk of 5 years and by normalizing the values of ${\theta }^{T}X$ to a risk score between 0 and 1, where $\theta$ and $X$ are the coefficientsand predictors, respectively. However, without the baseline hazard we are unable to assess calibration and will report discrimination performance only [54].

In addition, no data source codes or vocabularies are provided for the predictors, so that predictors needed to be retrieved from the medical terms.

#### Nori model

The Nori model did not explicitly report the time-at-risk. As with the Mehta model, we are still able to validate the model for an estimated time-at-risk of 5 years.

The paper provides ICD-9-CM codes for diagnoses and CPT-4 codes for procedures that are used as predictors in the final model. The OMOP-CDM uses CPT-4 as the standard vocabulary for procedures and SNOMED CT for diagnoses. However, a mapping table from ICD-9-CM to SNOMED CT is available. Therefore, we could retrieve predictors using exact code definitions for the Nori model.

A characteristic of the Nori model was a complex target population definition with multiple entry events and various observation windows. Given a written definition and graphical representation (Fig. 1 in original paper) of the target population, validation was notably more difficult than for the other models [13].

### External validation performance

**Fig. 2:**
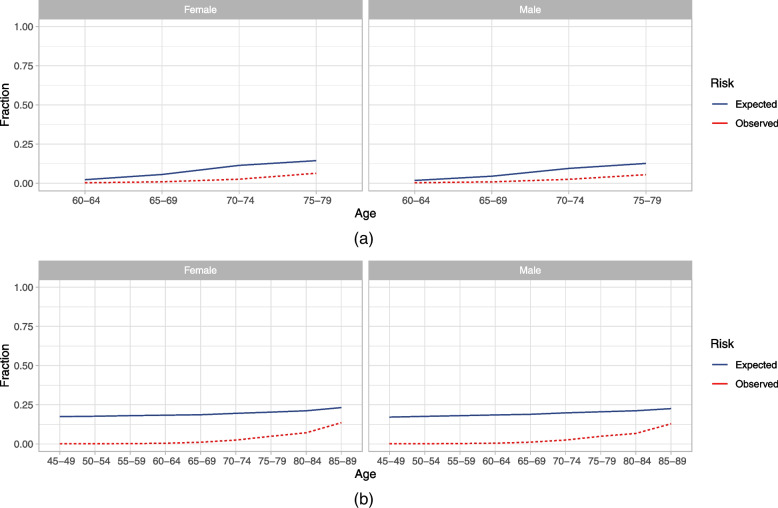
Round-trip calibration presented as observed versus expected risks across sex and age for non re-calibrated models: **a** Walters’ Dementia Risk Score on IMRD; **b** Nori’s ADRD prediction model on OPEHR. The shaded are presents the 95% confidence interval of the expected risk

Table 5 provides the discrimination and Table 6 the recalibration performance of the validated models. Calibration and re-calibration in terms of the E_avg_ was only assessed if the model’s authors provided the baseline risk, for example in the form of the intercept or baseline hazard. In Fig. 2 we present “round-trip” calibration as observed versus expected risks.

Walters’ Dementia risk score performed best during its development on THIN and worst after model validation on CPRD, MDCR, and IMRD. Interestingly, IMRD, which incorporates THIN, presents the best approximation of the development data and still shows a significant performance deterioration for the round-trip. Figure 2a shows the Walters model round-trip calibration of the original Walters model on IMRD indicating moderate agreement between observed and predicted risk for the entire target population.

Mehta’s RxDx-Dementia Risk index performed best during development on CPRD and almost equally well in the three primary care databases CPRD, IPCI, and IMRD.

Nori’s ADRD dementia prediction model performed best during development on OptumLabs and almost equally well in the remaining data sources. Interestingly, the round-trip performance on OPEHR was the worst. In Fig. 2b we learn that the model overpredicts the round-trip risk in the target population of CPRD.

Almost all models show improvements of the E_avg_ after recalibration (Table 5). Recalibration for the Mehta model was not assessed because no baseline hazard was provided.

**Table 5:** Internal and external discrimination performance in AUROC of externally validated models. The round-trip performances for each model are presented in the shaded cells

Model	Development database	MDCR	IQGER	OPSES	OPEHR	CPRD	IPCI	IMRD
Walters	0.84 (THIN)	0.69 (0.69–0.69)^a^	0.75 (0.75–0.75)^a^	0.74 (0.74–0.74)^a^	0.73 (0.73–0.73)^a^	0.67 (0.66–0.67)^a^	0.76 (0.75–0.77)^a^	**0.68 (0.68–0.69)**^a^
Mehta	0.81 (CPRD)	0.69 (0.69–0.70)	0.72 (0.71–0.72)	0.71 (0.70–0.71)	0.73 (0.73–0.73)	**0.79 (0.78–0.80)**	0.78 (0.76–0.80)	0.79 (0.78–0.80)
Nori	0.69 (Optum)	0.66 (0.66–0.67)	0.67 (0.66–0.68)	0.67 (0.66–0.68)	**0.62 (0.62–0.63)**	0.68 (0.67–0.69)	0.64 (0.62–0.67)	0.68 (0.68–0.69)

^a^Discrimination AUROC (95% confidence intervals)

**Table 6:** External calibration and recalibration performance in E_avg_ of externally validated models. Calibration of Mehta’s RxDx-Dementia Risk Index was not assessed due to missing baseline hazard

Model	MDCR	IQGER	OPSES	OPEHR	CPRD	IPCI	IMRD
Walters	0.060 (0.002)^a^	0.025 (0.011)	0.064 (0.011)	0.057 (0.032)	0.073 (0.015)	0.024 (0.011)	0.065 (0.001)
Mehta	-	-	-	-	-	-	-
Nori	0.164 (0.001)	0.142 (0.001)	0.258 (0.001)	0.170 (0.001)	0.198 (0.001)	0.790 (0.0002)	0.19 (0.001)

^a^Calibration E_avg_ (recalibrated E_avg_)

## Discussion

We assessed reporting of published dementia prediction models and found shortcomings in reporting essential information that would allow for full model validation.

Our results showed that while reporting was complete for some criteria such as target and outcome definitions, reporting of statistical analysis criteria is mostly insufficient to fully validate the dementia prediction models. Moreover, our external validation of three selected models showed that even if reporting was sufficient for model retrieval, it does not guarantee that external validation becomes non-trivial, because predictors had to be present, and inclusion and exclusion criteria of target and outcome had to be generalizable to other data sources. Performance across external data sources showed substantial differences in discrimination performance as compared to the reported development performance.

### Model reporting

All studies reported the target population and the outcome. However, only 22 of 59 models reported the index date. The problem arises that although it is clear for which (sub) population a risk model is meant to be used, it often remains unclear at what point in time the model is to be applied. A better solution for choosing an overall index date is using a visit or a condition diagnosis, which are associated with an individual date per patient. Additionally, a visit or a diagnosis date most likely involves interaction with a healthcare provider who is qualified to apply a model and interpret its results.

The time-at-risk is anchored to the index date and determines during which time the predictions of a model are valid. There were 42 of 59 models that explicitly reported the time-at-risk, while for the remaining studies it was unclear. Some studies would use the full follow-up of each individual patient, however, it remains unclear what the valid time frame is following the index date, for which reason these models cannot be applied reliably.

A majority of the studies reported predictor definitions or at least names that can be interpreted to replicate a predictor. However, only 21 of 59 models provided the time window in which the predictor is measured. Predictor definitions or descriptions without time window are not useful for non-demographic predictors. It could make a significant difference whether a predictor was recorded recently or 20 years in the past. More importantly, the validated model should match the original model settings, which cannot be achieved if predictor time windows are not reported. In addition, only 17 of 59 models provided a partial model, for example only coefficient values, and 9 of 59 models provided the full model. Therefore, our results suggest that while population settings are moderately well reported, there is lack of reporting statistical analysis settings, which in many cases makes external validation impossible.

Calibration is essential to assess if a model underestimates or overestimates outcome risk in an external population. Original calibration can be computed, if the intercept/baseline hazard or similar baseline risk parameters are reported. Only 9 of 59 models provided this information. Recalibration should generally be done, which yields best results if the original baseline risk is known.

#### Walters model

The performance of the Walters model in the external databases was good.

The “round-trip” performance after validating on IMRD is low compared to the development performance on THIN, yet agrees with the performance on CPRD, which is also a UK primary care database of similar structure. The reason for this performance deterioration is not immediately evident. Possible causes may be the entry events that could not be replicated, the visit entry event we defined ourselves, the missing social deprivation predictor, or inaccuracy when matching the literal predictor names to Read codes. Moreover, the conversion of the IMRD database to the OMOP CDM may have additionally introduced medical term inaccuracies.

#### Mehta model

The Metha model saw a drop off in performance across all external data sources. The “round-trip” performance after validating on CPRD was 0.79 as compared to 0.81 during development. This model was explicitly reported, which made validation easier, for example predictors were provided in the form of code lists. We had to assume the missing time-at-risk to be 5 years, which is a value commonly used across the reviewed models. Due to the good performance on CPRD, we are confident that the model is mostly well retrieved, despite not having the baseline hazard. However due to incomplete model specification, calibration is unknown. For reporting, we recommend that authors of Cox proportional hazard models obtain an approximation of the baseline hazard, for example through method presented by Royston et al. [54, 55].

Generally, a model that is not completely reported should not be considered for clinical use. In this case, the 5-year time-at-risk appears to work well since we based it on the design choice of other reviewed dementia models. However, instead of retrieving an incomplete model we suggest to take explicit target cohort, outcome or predictor definitions as a starting point to build new models directly on the validation databases.

#### Nori model

The Nori model was the lowest performing model with a 0.69 AUC during development. This dropped to 0.62 on OPEHR, after the “round-trip” while maintaining good calibration (Fig. 2b). The target population definition appeared complex, with four different cohort entry events causing difficulties during validation. Validation would have benefitted from a more verbose and systematic presentation of such a complex target population.

### Implications

The lack of external validation in dementia prediction literature can to some extend be attributed to the insufficient reporting of models. Models should be developed with external validation in mind. This could for example mean to report all aspects of the model explicitly. Such transparency is best achieved programmatically through code lists and underlying logic rather than literal descriptions, for example by providing a full description of the model (development) in code, ideally against a common data model. This approach will likely eliminate ambiguity as a source of error. For example, Nori’s ADRD prediction model uses two variables named “Diabetes Mellitus”, which originate from ICD9CM codes 250.00 and 250.02. If these codes were not provided by the authors, it would not have been possible to verify that the former code specifies “not stated as uncontrolled” and the latter “uncontrolled”.

Development choices should not rely on properties unique to the development database, e.g., the Walters model contained criteria to define the target population and predictors that did not exist in the external data sources, for example the cohort entry event “one year following new registration with a THIN practice”.

In general, authors should avoid uncommon predictors during model development if the model is meant to be applied in external healthcare settings. Instead of building a single model with multiple, complex cohort entry events, it can be beneficial to build a model for each entry event, which may be easier to interpret and validate. The Nori model suffered from this problem as it had a complex target population definition with multiple entry events. Defining the time-at-risk window is crucial to indicate in which time window a model’s predictions are valid. Using the full follow-up of a population is not a valid approach, as follow-up can vary per person.

Recalibration showed improvements in the E_avg_ across most models and databases, however, this can only be observed when the intercept or baseline hazard is reported. To perform recalibration in an external setting, an annotated dataset is required. If such a dataset is available, the question arises, whether developing a new model altogether, potentially using definitions from existing models, may be an even better approach. Recalibration performed during our external validations shows improvements, but if no intercept/baseline hazard is reported possible recalibration improvements cannot be assessed.

Recalibration needs to be performed, if discrepancy between expected and observed risk is large, which can be assessed through visualizations such as Fig. 2.

A systematic review by Collins et al. concluded that also most external validation studies were characterized by poor design, inappropriate handling and acknowledgement of missing data, and calibration performance often being omitted from the publication [56]. We believe that explicitness in reporting by providing computer executable code based on which the development, but also the external validation has been performed, will facilitate the implementation of a prediction model in clinical practice to ultimately improves patient outcomes. The TRIPOD statement on which our reporting criteria are based can serve as a valuable resource towards better reporting [24].

### Limitations

We reviewed studies for reporting criteria to the best of our ability, however, due to vague descriptions, that leave room for interpretation, our general approach was to consider criteria as not reported once uncertain. Moreover, this study is purely methodological and assesses the quality of model reporting in existing dementia prediction literature. We did not assess the clinical usefulness of any of the validated models, nor do we endorse any of the models for clinical use.

The “round-trip” is meant to approximate the performance of the retrieved model on the original development data. However, over time the composition of people and other records in the databases may have changed for various reasons. For example, on October 1, 2015, the Centers for Medicare & Medicaid Services (CMS) in the Department of Health and Human Services (HHS) in the United States coordinated the transition from the ICD-9 code sets to ICD-10 codes. An increased number of codes in ICD-10 can manifest in discrepancies for data analyses before and after this transition. Equally, regulatory changes can result in drug or procedure records becoming available or unavailable in the data from one day to the other.

Moreover, databases were mapped to the OMOP CDM, which eliminates the need for the model to be adjusted for different database source codes. However, the accuracy of this mapping may have negatively impacted the round-trip performance of each model on their respective development database.

## Conclusion

Many dementia risk prediction models have been developed, but only a handful have been externally validated [3, 4]. We reviewed 35 studies that proposed a total of 59 dementia risk models. We observed that reporting is mostly insufficient to fully externally validate published dementia prediction models, and therefore, it is uncertain how well these models would work in other clinical settings. In addition, we externally validated three existing dementia prediction models and encountered difficulties beyond our reporting criteria, such as ambiguous cohort or predictor definitions. We emphasize the importance of following established guidelines for reporting clinical prediction model. We recommend that reporting should be more explicit and have external validation in mind if the model is meant to be applied in different settings.

## Supplementary Information

**Additional file 1: Appendix A.** Dementia prediction literature review.

## Data Availability

The Optum and IBM MDCR data that support the findings of this study are available from IBM MarketScan Research Databases (contact at: http://www.ibm.com/us-en/marketplace/marketscan-research-databases) and Optum (contact at: http://www.optum.com/solutions/data-analytics/data/real-world-data-analytics-a-cpl/claims-data.html) but restrictions apply to the availability of these data, which were used under license for the current study, and so are not publicly available. Due to ethical concerns, supporting data cannot be made openly available for the CPRD, IMRD, IPCI and Iqvia Germany datasets. The validated models that support the findings of this study are available in a study package on the mi-erasmusmc GitHub repository (https://github.com/mi-erasmusmc/EmcDementiaModelValidation). The study package requires the OHDSI environment (https://github.com/ohdsi), notably the Patient-Level Prediction framework (https://github.com/OHDSI/PatientLevelPrediction). More information can be found on the network homepage (https://OHDSI.org).

## Abbreviations

ADRD: Alzheimer’s disease and related dementias
OHDSI: Observational Health Data Sciences and Informatics
OMOP: Observational Medical Outcomes Partnership
PROGRESS: Prognosis Research Strategy
TRIPOD: Transparent reporting of a multivariable prediction model for individual prognosis or diagnosis
PLP: Patient-level prediction R-package framework
AUROC: Area under the Receiver Operating Characteristic curve

## Declarations

The New England Institutional Review Board determined that studies conducted in Optum, IBM MDCR, Iqvia Germany are exempt from study-specific IRB review, as these studies do not qualify as human subjects research.

IMRD (study reference 21SRC025), CPRD (study reference 22_001724) and IPCI (study reference 2/2022) had institutional review board approval for the analysis, or used deidentified data, and thus the analysis was determined not to be human subjects research and informed consent was not deemed necessary at any site. IMRD incorporates data from THIN, A Cegedim Database. Reference made to THIN is intended to be descriptive of the data asset licensed by IQVIA.
